# The relationship between social media addiction levels and happiness levels among healthcare professionals

**DOI:** 10.3389/fpsyg.2026.1886635

**Published:** 2026-07-10

**Authors:** Gulseren Citak Tunc, Büşra Köse

**Affiliations:** 1 Department of Psychiatric Nursing, Faculty of Health Sciences, Bursa Uludag University, Bursa, Türkiye; 2 Department of Nursing, Institute of Health Sciences, Bursa Uludag University, Bursa, Türkiye

**Keywords:** addiction, happiness, healthcare professionals, mental health, social media addiction

## Abstract

**Background and aims:**

This study was conducted cross-sectionally using a descriptive-correlational approach. Study aimed to examine the relationship between healthcare professionals ‘social media addiction and their level of happiness.

**Methods:**

The data was collected via a survey of healthcare professionals working at a public hospital in a major city in western Turkey between November 1 and December 31, 2024. IBM SPSS 26.0 software was used for data analysis.

**Results:**

The study included 225 healthcare professionals (65.8% female, 34.2% male), with an average age of 33.11 ± 9.07. The average daily social media usage time was 2.96 ± 1.49 h. Healthcare professionals had moderate levels of happiness (24.29 ± 4.07) and social media addiction (14.30 ± 4.81). Happiness scores did not differ significantly across sociodemographic characteristics (*p* > 0.05). However, social media addiction scores were significantly higher among women and single participants (*p* < 0.05). Pearson correlation analysis revealed that happiness score was positively correlated with age and years of work experience, and negatively correlated with social media addiction (*p* < 0.05). Social media addiction was negatively correlated with age and years of work experience, and positively correlated with daily social media usage time (*p* < 0.001). In the multiple linear regression analysis, the model was significant (*F* = 5.35; *p* < 0.01), and the social media addiction score explained 9% of the variance in happiness scores (R^2^ = 0.09).

**Conclusions:**

Only social media addiction is a significant predictor of happiness. The findings indicate that as social media addiction increases, happiness levels decrease.

## 1 Introduction

Technology has become an indispensable part of modern life. In particular, over the past decade, there has been a significant increase in the amount of time spent on social media platforms. Individuals use social media for various purposes such as communication, information gathering, sharing, self-expression, entertainment, shopping, and leisure activities (Diker, 2021). However, increased social media use can lead to problematic use and addictive behavior in some individuals. Social media addiction (SMA) is defined as overindulgence and compulsive use of social media platforms (Zivnuska et al., 2019).

Addictive behavior is defined as a person directing their attention toward an activity, substance, or behavior to such an extent that it causes them to neglect other areas of their life and results in physical, mental, or social harm (American Psychiatric Association, 2013). The concept of addiction, traditionally associated with substances, has expanded over time in the digital age to include behavioral addictions (Mosto et al., 2026). Social media addiction is characterized by excessive use of social media, loss of control over access, feelings of dissatisfaction, and allocating a substantial amount of one’s time and energy to social media (Bottaro et al., 2025). It is considered a behavioral addiction due to symptoms such as withdrawal, tolerance, relapse, and mood changes (Kesici and Tunç, 2018).

The global data reveals the prevalence of social media usage. According to the 2025 Global Overview Report, 5.24 billion people worldwide use social media, representing 63.9% of the global population. The average daily social media usage time is 2 h and 21 min. In Turkey, the social media usage rate is 66.7%, and the daily social media usage time is approximately 2 h and 43 min (We Are Social, 2025). The increase in time spent in the digital environment also raises the risk of social media addiction.

The literature indicates that SMA is influenced by attachment styles (Eichenberg et al., 2024), personality traits (Sheldon et al., 2021), sociodemographic variables (Hylkilä et al., 2025), and psychosocial factors (Jahagirdar et al., 2024). Research involving healthcare professionals has shown an increased risk of internet and SMA (Prasetya and Wardani, 2023). Taken together, these findings indicate that SMA warrants greater attention. Studies among adults working in diverse professional fields have shown high levels of social media addiction, indicating that the problem is not confined to younger age groups (Kong et al., 2024).

Happiness is an indicator of well-being that encompasses an individual’s subjective evaluation of their life satisfaction. Personality traits, self-esteem, life satisfaction, and sociability are among the determinants of happiness. Social media use has both direct and indirect effects on happiness. Excessive and problematic social media use may negatively affect happiness through deprivation and mood changes (Meynadier et al., 2025). Furthermore, it is stated that social media networks can cause individuals to question their own reality and may affect life satisfaction (Çat et al., 2021).

According to the Turkish Statistical Institute’s [TUIK] Life Satisfaction Survey, 52.7% of individuals aged 18 years and older describe themselves as happy (Türkiye İstatistik Kurumu (TUIK), 2023). Happiness is related to job satisfaction, working conditions, income level, and mental health (Huang et al., 2023). Previous studies have reported that social media use can lead to mental health problems and exacerbate existing mental health problems (Karim et al., 2020). It has been reported that SMA among healthcare professionals is positively associated with burnout (Luo et al., 2022), while excessive social media use is linked to anxiety, depression, and substance use (Prasetya and Wardani, 2023).

Maintaining the mental well-being of healthcare professionals and ensuring the balanced use of social media are important not only for individual health but also for the quality of healthcare services. In this context, it is important to examine the relationship between social media addiction and happiness among healthcare professionals.

This study aimed to examine the relationship between healthcare professionals ‘social media addiction and their level of happiness. Additionally, the relationship between social media addiction and happiness levels with sociodemographic characteristics (age, gender, professional title, years of work experience, and daily social media usage time) was evaluated. Social media addiction, on the other hand, is the primary independent variable of the study and constitutes the main research question. The study tested whether social media addiction is a significant predictor of happiness.

Research Hypothesis

*H1*: Healthcare professionals’ social media addiction and happiness levels significantly differ according to their sociodemographic characteristics.

*H2*: There is a significant relationship between healthcare professionals’ social media addiction, happiness, and sociodemographic characteristics.

*H3*: Social media addiction is a significant predictor of happiness.

## 2 Materials and methods

### 2.1 Study design

This study was conducted using a cross-sectional and descriptive research design. The data in the study were collected through face-to-face questionnaires.

### 2.2 Participants

The study population consisted of 237 healthcare professionals employed in a public hospital. The sample size was calculated using the known population sample size equation (Taherdoost, 2017).

$$n=\frac{\left(N.t2.p.q\right)}{d^{2}.(N-1)+t2.p.q}$$

In the sample size calculation, the margin of error (d) was set at 0.05, the t value corresponding to the confidence level was 1.96, and the *p* value and q value were both assumed to be 0.50. Based on these calculations, the required sample size was determined to be 147. It was aimed to reach the entire study population, and the study was conducted with a total of 225 healthcare professionals in December 2024.

### 2.3 Ethical approval

Prior to the initiation of the study, ethical approval was obtained. The study was conducted in accordance with the principles of the Declaration of Helsinki. All participants were informed about the purpose of the study, and informed consent was obtained the information statement presented at the beginning of the questionnaire.

### 2.4 Measures

#### 2.4.1 Sociodemographic characteristics

A sociodemographic information form developed by the researchers was designed to determine the demographic and professional characteristics of healthcare professionals. The form included questions regarding participants’ age, gender, marital status (married/single), professional title (physician, nurse, healthcare technician, etc.), education level, income level, years of work experience, and daily social media usage time (hours).

#### 2.4.2 Bergen Social Media Addiction Scale (BSMAS)

The Bergen Social Media Addiction Scale was developed by Andreassen et al. (2017) and consists of six items with a unidimensional structure. The scale is rated on a five-point Likert scale (1 = Very rarely, 5 = Very often), with total scores ranging from 6 to 30. The original scale demonstrated good internal consistency, with a Cronbach’s *α* coefficient of 0.88. Results of the confirmatory factor analysis indicated that the one-factor structure showed acceptable model fit (Andreassen et al., 2017). Although no definitive cutoff score has been established for the scale, obtaining a score of 3 or higher on at least four of the six items may be interpreted as indicating a high level of social media addiction. The Turkish adaptation of the scale was conducted by Demirci (2019), who reported a Cronbach’s *α* coefficient of 0.83. In the present study, the Cronbach’s α coefficient was found to be 0.75.

#### 2.4.3 Oxford happiness questionnaire–short form (OHQ-SF)

The Oxford Happiness Questionnaire–Short Form was developed by Hills and Argyle (2002). The original scale has a unidimensional structure and consists of eight items, with a reported Cronbach’s *α* = 0.93. The short form was adapted into Turkish by Doğan and Çötok (2011) and consists of seven items. The scale is rated on a five-point Likert scale, with total scores ranging from 7 to 35. Higher scores indicate higher levels of happiness. Items 1 and 7 are reverse-coded. The Cronbach’s α coefficient of the Turkish version was reported as 0.74. In the present study, Cronbach’s α was found to be 0.74.

### 2.5 Procedure

Before the data collection phase, a questionnaire form was developed by the researchers. The data collection process was conducted face-to-face in November 1 and December 31 2024. The data was collected during the time healthcare workers were at the hospital and was entirely voluntary. Participants were informed about the purpose of the study, and the questionnaire was administered after obtaining written informed consent. A total of 227 surveys were collected; however, two of them were excluded from the study because the scale questions were not answered. Thus, analyses were conducted using 225 valid questionnaires, resulting in an effective response rate of 94.93%; only questionnaires with complete responses to all items were included.

### 2.6 Statistical analysis

IBM SPSS 26.0 software was used for data analysis. The demographic characteristics of healthcare professionals were examined using descriptive statistics (frequency, percentage, mean, standard deviation, etc.). The relationship between OHQ-SF and BSMAS scores was examined using Pearson correlation analysis. Scale scores were compared according to gender and marital status using the independent groups *t*-test, while comparisons according to professional title, education level, and income level were made using one-way analysis of variance (ANOVA). Variables predicting OHQ-SF scores were examined using multiple linear regression analysis. The variables included in the multiple linear regression model were selected based on the research objectives and hypothesis, as well as theoretical evidence from the literature. In this context, age, gender, income level, and social media addiction score were included in the model as independent variables. Before to the regression analysis, the model assumptions were evaluated. Multicollinearity was assessed using tolerance and variance inflation factor (VIF) values, while the independence of error terms was evaluated using the Durbin–Watson statistic. In all analyses, the level of statistical significance was set at *p* < 0.05. The normality of the data distribution was checked by considering skewness and kurtosis values (±1.5).

## 3 Results

A total of 225 healthcare professionals participated in the study. The mean age of the participants was 33.11 ± 9.07 years (Range: 21–62), and 65.8% were female (*n* = 148). Of the participants, 70.7% (*n* = 159) held a bachelor’s degree, 61.3% (*n* = 138) were married, 43.1% (*n* = 97) were nurses, 20.9% (*n* = 47) were physicians, 20.4% (*n* = 46) were healthcare technicians, and 16.6% (*n* = 35) had other professional titles. 56% of participants (*n* = 126) stated that their income was equal to their expenses. The mean daily social media usage time was determined to be 2.96 ± 1.49 h (Min 1.00 Maks:10.00). The mean years of experience was 10.32 ± 9.352 years (Min:1 Maks:39).

The analyses revealed that happiness scores did not differ significantly according to gender, education level, marital status, professional groups, and income level (*p* > 0.05). However, social media addiction scores showed significant differences according to gender and marital status. Women had significantly higher social media addiction scores than men, and single participants had significantly higher scores than married participants (*p* = 0.011 and *p* = 0.001, respectively) (Table 1).

**Table 1:** Comparison of social media addiction and happiness score averages according to the characteristics of healthcare professionals (*n* = 225).

Characteristic	Total (%) (*n* = 225)	OHQ-SF mean score	*p*	t/F	BSMAS mean score	*p*	t/F	Mean ± SD	Range (min–max)
Gender									
Female	148 (65.8)	24.43 ± 4.13	0.480	−0.708*	14.89 ± 4.89	0.011	−2.579*		
Male	77 (34.2)	24.02 ± 3.37			13.16 ± 4.46				
Education level									
Associate degree	36 (16)	24.77 ± 4.40	0.375	0.986**	14.38 ± 5.15	0.121	2.129**		
Bachelor’s degree	159 (70.7)	24.05 ± 4.05			14.59 ± 4.81				
Graduate degree	30 (13.3)	25.0 ± 3.79			12.63 ± 4.18				
Marital status									
Married	138 (61.3)	24.44 ± 4.16	0.471	0.721*	13.46 ± 4.76	0.001	−3.365*		
Single	87 (38.7)	24.04 ± 3.94			15.63 ± 4.61				
Professional groups									
Physician	47 (20.9)	23.72 ± 3.62	0.644	0.557**	13.70 ± 4.31	0.755	0.397**		
Nurse	97 (43.1)	24.27 ± 4.18			14.60 ± 5.17				
Healthcare technician	46 (20.4)	24.47 ± 4.24			14.41 ± 4.93				
Other (Physiotherapist, dietitian, dentist, pharmacist, midwife)	35 (16.6)	24.85 ± 4.21			14.11 ± 4.31				
Income level									
Less than expenses	40 (17.8)	23.90 ± 3.99	0.796	0.229**	14.00 ± 4.55	0.222	1.516**		
Equal to expenses	126 (56)	24.39 ± 4.22			13.96 ± 5.05				
Greater than expenses	59 (26.2)	24.33 ± 3.86			15.23 ± 4.39				
Age								33.11 ± 9.07	21–62
Years of work experience								10.32 ± 9.352	1–39
Daily social media usage time (hour)								2.96 ± 1.49	1–10

*t test; **ANOVA.

Scores of the main variables are presented in Table 2. The Oxford Happiness Questionnaire–Short Form, consisting of 7 items, ranges from 7 to 35. In this study, the mean happiness score of healthcare professionals was 24.29 ± 4.07.

**Table 2:** Scores of the main variables.

Variable	Items	Score range	Scale score (mean ±SD)
Oxford Happiness Questionnaire - Short Form (OHQ-SF)	7	7–35	24.29 ± 4.07
Bergen Soscial Media Addiction Scale (BSMAS)	6	6–30	14.30 ± 4.81

The Bergen Social Media Addiction Scale consists of 6 items and ranges from 6 to 30. In this study, the mean social media addiction score of healthcare professionals was 14.30 ± 4.81. The mean daily social media usage time was 2.96 ± 1.49 h (Range: 1.00–10.00). The mean age of the participants was 33.11 ± 9.07 years (Range: 21–62). The mean years of experience was 10.32 ± 9.352 years (Range: 1–39) (Table 2).

According to the results of the Pearson correlation analysis, happiness scores were positively and weakly correlated with age (*r* = 0.143, *p* < 0.05) and years of work experience (*r* = 0.143, *p* < 0.05). Social media addiction scores were negatively correlated with happiness (*r* = −0.273, *p* < 0.001), age (*r* = −0.388, *p* < 0.001), and years of work experience (*r* = −0.344, *p* < 0.001), and positively correlated with daily social media usage time (*r* = 0.436, *p* < 0.001) (Table 3).

**Table 3:** Correlation between happiness scores and social media addiction scores between the key study variables.

Scales		Age	Years of work experience	Daily social media usage time (hours)	Bergen Social Media Addiction Scale (BSMAS)
Oxford Happiness Questionnaire - Short Form (OHQ-SF)	*r*	0.143*	0.143^*^	−0.038	−0.273**
	*p*	0.032	0.031	0.575	<0.001
Bergen Social Media Addiction Scale (BSMAS)	*r*	−0.388**	−0.344^**^	0.436^**^	
	*p*	<0.001	<0.001	<0.001	

*r* = Pearson correlation analysis. *p < 0.05, **p < 0.001.

According to the multiple linear regression analysis, only the social media addiction score was a statistically significant predictor of happiness (*p* < 0.001). A negative relationship was observed between social media addiction and happiness scores, with a 95% confidence interval ranging from −0.349 to −0.113. These findings indicate that as the level of social media addiction increases, the level of happiness decreases (Table 4).

**Table 4:** Multivariate linear regression analysis for variables affecting happiness scores.

Variables	Unstandardized coefficients		Standardized coefficients	*t*	*p*	95.0% CI	
	B	Std. error	Beta			Lower bound	Upper bound
(Constant)	25.970	1.658		15.660	<0.001	22.702	29.239
Age	0.028	0.032	0.061	0.867	0.387	−0.035	0.090
Gender	0.923	0.567	0.108	1.627	0.105	−0.195	2.041
Income level	0.426	0.601	0.046	0.709	0.479	−0.758	1.611
BSMAS	−0.231	0.060	−0.273	−3.862	<0.001	−0.349	−0.113

Multivariate linear regression analysis, R^2^ = 0.09, F = 5.35, *p* < 0.001. The regression model assumptions were met. No multicollinearity was observed (VIF = 1.018–1.205; tolerance = 0.830–0.983), and the Durbin–Watson coefficient was found to be 1.827. Female gender and high income are coded as 1 in the regression analysis.

## 4 Discussion

The findings indicate that social media addiction affects happiness among healthcare professionals. The findings are discussed under four main themes.

### 4.1 The relationship between social media addiction levels and sociodemographic characteristics among healthcare professionals

In the present study, no significant relationship was found between healthcare professionals age and their social media addiction levels, and the overall level of social media addiction was moderate. There are studies conducted on healthcare professionals using different measurement tools related to SMA. Results of a study conducted among nurses in China using the Social Network Service Addiction Scale (SNSAS), which reported that nurses with shorter work experience had the highest levels of social media addiction compared to other groups (Kang et al., 2025). Another study conducted among also indicates that social media addiction decreases with age (Balcı et al., 2020). In our study, young healthcare professionals and those with more work experience had similar levels of social media addiction. This result can be attributed to similar working conditions and the widespread use of digital technologies across all age groups, as well as sample characteristics and cultural factors.

In our study, no significant difference was found in social media addiction levels according to gender among healthcare professionals. Similar findings have been reported in studies conducted among university students (Eladl and Musawi, 2021), young adults (Avcı and Ünal, 2024), and healthcare professionals (Balcı et al., 2020). However, while a study conducted in Romania reported that women (Stănculescu and Griffiths, 2022) had higher SMA levels, a study in Sweden reported that men (Dorrestein et al., 2025) had higher SMA levels. Meta-analysis results indicate that personality traits and psychosocial factors may be determinants of SMA (Kayış et al., 2016; Pontes et al., 2018). Therefore, the absence of gender differences in our study may be explained by the more dominant role of individual and psychosocial characteristics. Additionally, the fact that social media use is equally accessible to both genders and that healthcare professionals have similar duties and responsibilities for both genders may have influenced this result.

According to the findings of this study, no significant differences were found in SMA levels according to education level and professional title. A recent study conducted on healthcare professionals reported a significant difference between social media addiction levels and education level (Karakoç et al., 2024). However, this study did not find a significant difference in regard to education level and professional title. This finding may be explained by the easy accessibility of social media across all education levels and professional titles, as well as its similar use for work-related purposes such as communication and information gathering.

### 4.2 The relationship between happiness levels and sociodemographic characteristics among healthcare professionals

The results of this study indicate that have moderate levels of happiness. This conclusion is consistent with studies conducted in Turkey (Kose et al., 2018; Solak and Yurttaş, 2025), Iran (Azar et al., 2025; Javanmardnejad et al., 2021), and China (Kang et al., 2025). A comparative study found that the happiness levels of those working in the healthcare sector were significantly lower than those working in the education and security sectors (Yıldız, 2024). Healthcare professionals are exposed to violence more frequently than employees in other sectors. A recent comparative study conducted with physicians in Turkey and Romania found that the rate of exposure to violence in healthcare services was 76% in Turkey and 44% in Romania (Ekinci et al., 2025). The findings on happiness in this study may also be related to the increasing trend of violence against healthcare professionals in Turkey.

No significant difference was found in happiness levels according to gender, education level and professional title. While similar findings have been reported in the literature (Balcı and Demir, 2018), some studies have indicated higher rates of depression in women (Aslan and Polat, 2024) and lower levels of occupational happiness among female healthcare professionals (Huang et al., 2023). Research findings also indicate that men report higher life satisfaction (Shahid et al., 2024). However, the absence of a significant gender-based difference in the present study suggests that the high workload, occupational stress factors, and widespread burnout in the healthcare sector may have mitigated the effects of gender-related differences.

A study conducted in Thailand reported that nurses had lower life satisfaction than doctors (Chairassamee and Chancharoenchai, 2025). There is also research indicating that people living in regions with higher levels of education are happier (Oishi and Gilbert, 2016). However, this study did not find a significant difference in regard to education level and professional title. This data from the study may be related to the study being conducted in a state hospital and to the homogeneity of the sample, as participants had similar institutional arrangements, working conditions, and job demands.

### 4.3 The relationship of social media addiction and happiness with sociodemographic characteristics

Spending more time on social media platforms among young adults may increase the risk of addiction. This situation can be explained by young individuals’ more active use of social media platforms, their faster adaptation to new applications, and their early exposure to digital technologies. Similar conclusions can be found in the literature (Balcı et al., 2020; Kang et al., 2025). Based on the findings, higher levels of addiction among younger employees highlight the role of age and years of work experience. A review of the literature supporting our findings reports that problematic social media use presents a higher risk profile, particularly among younger age groups (Hylkilä et al., 2025). Young people and university students are identified as important risk groups in terms of social media addiction (Liang et al., 2024). In a study conducted with individuals aged 13–21, it was reported that 72% of participants had high levels of social media addiction (Victor et al., 2024). However, there are also studies reporting no significant relationship between age and social media addiction (Shabahang et al., 2022). This result is thought to be related to characteristics specific to the study sample.

There is no clear agreement in the literature regarding the relationship between age and happiness (Bartram, 2024). Studies conducted in European countries have determined that happiness increases after the age of 50 and then shows a tendency to decrease again in later years (Becker and Trautmann, 2022). Although our study found a low level of positive correlation between age and happiness, this relationship was not significant in the multiple linear regression model. This can be explained by the multidimensional nature of happiness and the mediating role of other psychosocial variables. Young adults aged 18–35, having been exposed to digital technologies at an early age (Odgers and Jensen, 2020), may contribute to more intensive social media use in this group. It has been reported that among individuals in the higher income brackets, happiness does not vary significantly with age and even peaks in middle age (Toshkov, 2022). A study indicates that young female healthcare professionals are prone to low happiness, while older female healthcare professionals have higher happiness and life satisfaction (Chairassamee and Chancharoenchai, 2025). Profile analyses conducted in the context of behavioral addictions emphasize that individual differences are determinative in the emergence of problematic social media behaviors (Packer and Flack, 2024).

As age increases, the relationship between age and happiness may be explained by factors such as reduced life-related uncertainties, a more organized lifestyle, increased prosperity levels, having a family, and stronger social support systems. Additionally, it is noted that mindfulness appears to function as a protective factor against SMA (Li et al., 2021). In this regard, it is assessed that the risk of social media addiction among healthcare professionals may be related not only to the age factor but also to individual and situational psychosocial characteristics. These may include individual factors such as coping styles, psychological resilience, perceived stress levels, and personality traits, as well as situational factors such as workload, burnout levels, and the availability of social support.

### 4.4 The relationship between social media addiction and happiness

One of the main findings of the study is that as social media addiction increases, happiness levels decrease. In addition, a positive relationship was found between daily social media usage time and social media addiction. This result indicates that spending more time on social media may increase the risk of addiction. A recent meta-analysis demonstrated that social media use is associated not only with social media addiction but also with depression and anxiety (Ahmed et al., 2024). This results is consistent with studies reporting that higher levels of social media addiction are associated with lower levels of happiness (Al-Samarraie et al., 2022; Çiftci and Yıldız, 2023), that social media addiction is positively associated with depression (Haand and Shuwang, 2020), and that individuals with social media addiction have lower levels of happiness and life satisfaction (Eladl and Musawi, 2021).

Studies conducted among healthcare professionals have similarly reported a positive relationship between social media addiction and depression (Jahagirdar et al., 2024). In addition, one study found that the prevalence of probable depression among participants was 46.4%, and that increases in job satisfaction were associated with significant decreases in both social media addiction and depression levels (Al et al., 2022). From the perspective of healthcare professionals, excessive social media use may have negative effects on professional performance and psychological well-being. A decline in professional performance may lead to outcomes such as reduced work productivity, problems with attention and concentration, a decrease in the quality of patient care, and an increased risk of errors.

Among the four predictor variables (age, gender, income level, and social media addiction), social media addiction accounted for 9% of the total variance in happiness. The confidence interval being entirely negative indicates a significant inverse relationship, whereby increasing levels of social media addiction are associated with decreasing happiness. This direction of relationship was consistent in both correlation and regression analyses. These results demonstrate that social media addiction is a significant predictor of happiness. Moreover, the significance of a single predictor within a multifactorial construct such as happiness underscores the strength of this relationship.

The study revealed that one of the determining factors of happiness among healthcare professionals is social media addiction. Prolonged use of social media is associated with depression, anxiety, and stress, and is considered a contributing factor to social withdrawal, low self-esteem, and feelings of inferiority (O’Reilly et al., 2019). Excessive and problematic social media use can therefore be regarded as a barrier to happiness among healthcare professionals. Happiness, as a dimension of mental health and well-being, also significantly impacts the quality of patient care and patient safety (Hall et al., 2016). In a recent study conducted in Turkey with employees from both the private and public sectors, social media addiction was found to be associated with time theft (Cohen and Özsoy, 2025). From this perspective, social media addiction may function as a “time thief” in the healthcare sector, potentially leading to organizational costs.

## 5 Conclusion and recommendation

The study found that healthcare professionals’ social media addiction and happiness levels were moderate. Happiness scores did not show significant differences according to sociodemographic characteristics. However, social media addiction scores were found to be significantly higher in women and single participants. A negative relationship was identified between social media addiction and happiness. Social media addiction was negatively associated with age and years of work experience, while it was positively associated with daily social media usage time. Among the four predictor variables explaining happiness scores (age, gender, income level, and social media addiction), social media addiction accounted for 9% of the total variance. Social media addiction among healthcare professionals is a significant risk factor that can lead to serious organizational costs in the healthcare sector and affect happiness. According to the research hypotheses, while H1 and H2 were partially confirmed, H3 was supported. Digital awareness–based preventive interventions should be implemented for younger healthcare professionals and those with limited professional experience. Furthermore, considering the negative impact of high levels of social media addiction on happiness, healthcare institutions may benefit from establishing in-house psychosocial support units.

### 5.1 Limitations

This study has certain limitations. This research is limited to healthcare personnel working at a state hospital in a major city located in western Türkiye. It is also limited in terms of causal inference due to the nature of the cross-sectional research design. It is also limited by the explanatory power of the regression model. Studies including healthcare professionals employed in different institutions and regions could provide broader generalisability. It is limited to the dimensions assessed by the measurement tools. Although the validity and reliability of the scales have been established, the reliance on self-reported data introduces the risk of response bias.

## Data Availability

The raw data supporting the conclusions of this article will be made available by the authors, without undue reservation.
